# Clinical and Surgical Challenges in Kidney Transplantation: Toward a Personalized Approach?

**DOI:** 10.3390/medicina58050604

**Published:** 2022-04-27

**Authors:** Evaldo Favi, Roberto Cacciola

**Affiliations:** 1 Kidney Transplantation, Fondazione IRCCS Ca’ Granda Ospedale Maggiore Policlinico, 20122 Milan, Italy; 2 Department of Clinical Sciences and Community Health, Università degli Studi di Milano, 20122 Milan, Italy; 3 Department of Surgery, KSAFH, Tabuk 71411, Saudi Arabia; rc.1968@icloud.com; 4 Department of Surgical Sciences, University of Tor Vergata, 00133 Rome, Italy

The continuously evolving practice of solid organ transplantation (SOT) in general and kidney transplantation (KT) in particular embodies the complexity of a composite, multi-step healthcare service. Consolidated knowledge and evidence-based care constantly mutate to adapt new scientific advances to old and emerging clinical or surgical challenges. Necessarily, the progressive changes in our praxis contribute to addressing the issues we face every day, from the well-established [1,2,3] to the uncommon ones [4], as well as to the unprecedented crisis caused by the ongoing SARS-CoV-2 pandemic [5].

This Special Issue of *Medicina* covers some of the critical aspects of our routine professional activity as surgeons and clinicians who are involved in the care of KT patients. Prof. Ponticelli produced an elegant review on new-onset diabetes mellitus after renal transplantation, offering a practical and ready-to-use guide for the management of this frequent yet difficult to handle medical complication [1]. Brescacin et al. diligently explored past and present knowledge on renal allograft vesicoureteral reflux, highlighting the limited evidence that is available and the need for a more rigorous approach in future studies [2]. In their contribution, Moroni and colleagues described how the fate of patients with ANCA-associated vasculitis and chronic kidney disease has changed dramatically over the last decade. Once recognized as poor transplant candidates, this complex group of recipients is now routinely transplanted with encouraging long-term recipient and allograft survivals [4]. In a provocative manuscript, Nardelli and coworkers questioned the preferential use of hemodialysis over peritoneal dialysis in patients awaiting a KT, promoting a more flexible (and perhaps less biased) vision of the two renal replacement modalities [3]. Lastly, the systematic review performed by the group of researchers from Tor Vergata University summarized current trends in the management of immunosuppression in KT recipients with SARS-CoV-2 infection [5].

A contextualized reading of the aforementioned articles clearly indicates the need for a “tailored approach” as a common denominator of present and future transplant-related activities. Such a commonly shared observation is not surprising, albeit rarely documented in a topic collection so consistently.

The practice of KT has historically found its very fundamentals in protocols and guidelines that have globally resulted in the best treatment for patients with end-stage renal disease [6]. Although it represents a strength in determining the superiority of KT over other renal replacement therapies, such conformity of clinical and organizational strategies may not fully reflect the priorities of patients as well as the challenges that we face daily as clinicians.

Beyond clinical and scientific challenges, the impact of the SARS-CoV-2 pandemic on intensive care unit services and staff has been immense, reflecting a well-documented disruption in organ donation activity and a consequent reduction in deceased donor transplants. Defining strategies aimed to promote living donation would represent the logical course of action for maintaining a sufficient volume of KT procedures, particularly in those countries that have witnessed a devastating first wave of COVID-19 [7,8]. It is also noticeable how the attitudes of patients on waiting lists might have been influenced by the pandemic. Our transplant candidates found themselves with remarkable concerns and dilemmas, to the extent that some of them have been refusing a long-waited organ [9] due to the increased vulnerability to SARS-CoV-2 caused by immunosuppression, which had to be balanced against the efficacy of vaccines and reliance on non-pharmaceutical interventions such as social distancing and isolation for an unforeseeable period of time [10]. All of this has also been occurring with the knowledge that prolonging the time on waiting list and increasing the risk of suspension is associated with more adverse outcomes [11].

Future challenges may be unpredictable. Nevertheless, facing the financial burden of a number of aspects of SOT will likely represent one of the most pressing. Such challenges may be revealed during unprecedented times of uncertainty caused by the pandemic paired with current geo-political climate and consequent financial instability. In particular, the efforts aimed to guarantee wider access to KT may be frustrated by the accurate analysis of the costs of waiting list maintenance, which contribute to increasing the actual costs of transplant services, as recently reported [12]. Undoubtedly, such observations cannot be applicable to all countries to the same extent. Different healthcare systems based on public or private funding may display remarkably different outcomes. The actual evolution of KT practices will be challenged by the health economics of the service. Structuring financial models that are aimed to safeguard the entirety of the process, including organ donation, retrieval, and transplantation in a single entity with scientific, professional, and financial inter-dependance, is of paramount relevance and has been successfully implemented at the national scale in some countries. Even more relevantly, the demonstrated cost effectiveness of KT as the “gold standard” of treatment for eligible patients may substantially contribute to the whole practice of SOT as well as to organ donation services in general.

Hopefully, the international transplant community will be able to respond to any future challenges that translate into patient and social welfare as well as to whole advances in a critical healthcare service to which we contribute.

## Data Availability

Not applicable.
